# Midterm Outcomes of Endovascular‐First Revascularization for Multilevel Peripheral Arterial Disease With Chronic Limb‐Threatening Ischemia: A Retrospective Study From Vietnam

**DOI:** 10.1155/ijvm/5524641

**Published:** 2026-08-05

**Authors:** Lam Van Nut, Trinh Vu Nghia

**Affiliations:** ^1^ Department of Vascular Surgery, Cho Ray Hospital, Ho Chi Minh City, Vietnam, choray.vn

**Keywords:** chronic limb-threatening ischemia, endovascular, multilevel peripheral arterial disease, revascularization

## Abstract

**Background:**

Peripheral arterial disease (PAD) with chronic limb‐threatening ischemia (CLTI) represents advanced systemic atherosclerosis and carries high risks of amputation and mortality. Multilevel arterial involvement is common and often requires revascularization of both inflow and outflow segments. However, midterm outcome data in Vietnam remain limited.

**Objectives:**

This study is aimed at evaluating midterm clinical outcomes and identifying factors associated with wound healing, all‐cause mortality, and major limb amputation after an endovascular‐first revascularization strategy for multilevel PAD with CLTI.

**Methods:**

This retrospective case series included 91 patients with multilevel PAD (≥ 2 anatomical levels according to TASC II) and CLTI (Rutherford 4–6) treated at a tertiary hospital in Vietnam from 2017 to 2019. Patients were followed for 24 months. Kaplan–Meier analysis was used to describe survival, limb salvage, and wound healing over 24 months. Univariable and multivariable logistic regressions were used to identify factors associated with fixed‐time binary outcomes within 24 months, including wound healing, mortality, and major limb amputation.

**Results:**

The mean age was 74.0 ± 11.6 years, and 82.4% were male. The ankle–brachial index improved significantly after intervention (0.2 vs. 0.5; *p* < 0.001). The mean follow‐up duration was 18.9 ± 8.3 months. At 24 months, mortality was 27.5%, major amputation occurred in 18.7% (limb salvage 81.3%), and 62.2% of patients with tissue loss achieved wound healing (median 5 months). Age ≥ 75 years and neutrophil‐to‐lymphocyte ratio ≥ 3.5 were independently associated with reduced wound healing. Wound healing was associated with lower mortality and amputation risk. Adjunctive open surgery reduced limb amputation.

**Conclusion:**

An endovascular‐first revascularization strategy for multilevel PAD with CLTI achieved acceptable midterm limb salvage and wound healing, although mortality remained considerable. Risk stratification and multimodal care are essential to improve outcomes.

## 1. Introduction

Peripheral arterial disease (PAD) is a condition in which atherosclerotic plaques cause narrowing or occlusion of the lower limb arteries, leading to reduced perfusion of downstream organs and related tissues. The most severe stage is progression to chronic limb‐threatening ischemia (CLTI), which occurs in approximately 10% of the PAD population [1]. In particular, among patients with CLTI, mortality and amputation rates are very high, resulting in a marked deterioration in quality of life and an increased burden of care [2–4]. Previous studies have reported that mortality may reach up to 20% within 6 months and exceed 50% at 1 year in patients with PAD and CLTI who do not undergo timely intervention and treatment [5, 6].

A typical feature of CLTI is its frequent association with multilevel arterial lesions [7], including the inflow segment (aortoiliac), the femoropopliteal segment, and the infrapopliteal segment. Global studies have also shown that patients with multilevel lower limb arterial lesions account for up to two‐thirds of those with severe symptoms [8]. These cases of chronic multilevel lower limb arterial occlusion and stenosis often present at a late stage of the disease; therefore, treatment is more complex, and the prognosis is much poorer compared with cases presenting only with intermittent claudication [9–11]. Revascularization in multilevel PAD presents many challenges due to complex lesions. Previously, surgical bypass was the standard treatment for these complex multilevel lesions. However, significant risks are observed in patients with multilevel PAD with CLTI and multiple comorbidities. With advances in medical technologies, endovascular revascularization is currently the commonly preferred strategy in many cases because of its minimally invasive nature. Previous studies have reported limb salvage rates of more than 80% at 1 year after intervention [10, 12, 13].

Although the success of endovascular intervention has demonstrated its principal role in the treatment of chronic multilevel lower limb arterial disease, research data remain limited in Asian populations, particularly in Vietnam. Most evidence on treatment outcomes is derived from Western studies, where patient characteristics are often different [14]. Therefore, this study is aimed at evaluating midterm clinical outcomes and identifying factors associated with wound healing, all‐cause mortality, and major limb amputation after an endovascular‐first revascularization strategy for multilevel PAD with CLTI at a tertiary referral hospital in Vietnam.

## 2. Material and Method

### 2.1. Study Design and Participants

This retrospective study included 91 patients with multilevel PAD who underwent an endovascular‐first revascularization strategy at the Department of Vascular Surgery, Cho Ray Hospital, a tertiary referral hospital in southern Vietnam, from December 2017 to December 2019.

All patients who had available follow‐up data within the 24‐month study period and met the inclusion criteria were enrolled. The study included patients who met the following conditions: (1) CLTI based on clinical presentation classified at admission as Rutherford Stage 4 (ischemic rest pain), Stage 5 (minor tissue loss/ulcer), or Stage 6 (major tissue loss/gangrene); (2) multilevel PAD confirmed on computed tomography angiography with reconstruction, defined as stenosis and/or occlusion involving at least two of the following three anatomical levels: aortoiliac, iliofemoral, and infrapopliteal, according to the TASC II [15] anatomical classification framework; and (3) treated with an endovascular revascularization strategy, with or without adjunctive open surgical procedures [16].

Patients were excluded if they had PAD due to vasculitis (Buerger disease), concomitant lower limb deep vein thrombosis, associated aortic pathology (aneurysm or dissection), other ipsilateral arterial diseases such as limb arterial aneurysm or thrombosed aneurysm, or arteriovenous fistula combined with chronic PAD, prior arterial intervention in the same limb, or no follow‐up visits after discharge and inability to be contacted after discharge.

### 2.2. Data Collection

Data were collected from medical records and included baseline variables such as demographic characteristics and risk factors, Rutherford classification at admission, use of antiplatelet therapy, and ASA physical status classification. The distribution and complexity of lesions at each anatomical level were classified according to TASC II. Procedural characteristics included the type of anesthesia, arterial access strategy, treated segments, balloon angioplasty and stenting, additional open revascularization procedures including femoral endarterectomy and Fogarty thrombectomy, and wound care or minor amputation procedures including wound debridement, digit amputation, and Chopart amputation. Postprocedural antiplatelet therapy was also recorded.

### 2.3. Primary Outcome Measures

The primary outcomes for this study were midterm survival, including wound healing time, evaluated through wound healing duration, limb salvage (amputation rates), and all‐cause mortality over a 2‐year follow‐up period. In accordance with the Society for Vascular Surgery guidelines, midterm outcomes were defined as those occurring from 12 months postprocedure [17]. To ensure the statistical integrity of the findings, a follow‐up loss rate of less than 5% was maintained, minimizing potential selection bias [18].

Outcomes were presented as percentages (%): (1) survival: recorded up to the last follow‐up visit; in cases of death, the place of death (home or healthcare facility) and cause of death were determined based on death certificates/medical documentation or clinical information obtained from relatives; (2) limb salvage/late limb amputation (major amputation above the ankle) and time to wound healing: assessed by vascular surgeons based on clinical and paraclinical findings (Doppler ultrasound/angiography) according to a standardized protocol; if late amputation occurred, the time from revascularization (months) was recorded. Wound healing was defined as complete epithelialization maintained for at least 14 days [19].

Clinical improvement was assessed using the 7‐grade Rutherford outcome classification. An increase of 1 grade on the Rutherford outcome scale was considered indicative of clinical improvement, including changes in Rutherford classification, changes in ankle–brachial index (ABI), and limb loss events [16]. Specifically, a score of +3 indicated absence of symptoms with healed wounds and ABI > 0.9, without amputation; +2 indicated improvement to Rutherford Stages 1–3 with at least one‐grade improvement and an increase in ABI > 0.1, without amputation; 0 indicated no significant change; negative scores indicated clinical deterioration, including an increase in Rutherford stage and/or a decrease in ABI > 0.1; among these, −3 included major amputation (at or above the ankle).

### 2.4. Study Procedure

All patients with chronic lower limb arterial disease presenting with CLTI (Rutherford 4–6) were screened and classified based on anatomical lesion severity on CTA as multilevel or single‐level disease. Single‐level cases were excluded from the study. Within the multilevel group, only patients who underwent endovascular revascularization (with or without adjunctive open surgery) were included in the analysis. Preoperative data were collected before intervention; after the procedure, patients were reassessed clinically and hemodynamically, and complications and early outcomes were recorded. After discharge, patients were followed at 1, 6, 12, and 24 months to evaluate clinical status, limb amputation, mortality, and wound healing; all data were completed and entered into statistical analysis.

### 2.5. Statistical Analysis

Data were entered using Epi Info 7.2, exported to spreadsheet format, and analyzed with R software (Version 4.3.2, R Foundation for Statistical Computing, Austria). Continuous variables are presented as mean ± standard deviation or median (IQR), and categorical variables as *n* (%). Comparisons between groups were performed using the *t*‐test or Mann–Whitney *U* test for continuous variables and the chi‐square test or Fisher exact test for categorical variables, as appropriate. ABI before and after intervention was compared using the Wilcoxon signed‐rank test for paired samples.

Time‐to‐event outcomes, including survival, limb salvage, and wound healing, were described using Kaplan–Meier estimates. For regression analysis, wound healing, all‐cause mortality, and major limb amputation were analyzed as fixed‐period binary outcomes within 24 months after revascularization. Factors associated with these outcomes were evaluated using univariable and multivariable logistic regression and reported as crude odds ratios and adjusted odds ratios with 95% confidence intervals. Variables with univariable *p* < 0.10 were entered into the multivariable model. Variables with a *p* value < 0.10 in univariable analysis were considered for inclusion in the multivariable logistic regression model. Statistical significance was defined as *p* < 0.05.

## 3. Results

### 3.1. Baseline Characteristics

A total of 91 patients underwent endovascular intervention for revascularization. The mean age was 74.0 ± 11.6 years, and the majority were male, accounting for 82.4% (75 patients). The general characteristics of the study population are shown in Table 1. Most patients had hypertension (52.7%) and diabetes (33.0%), and only two patients were smokers (2.2%). At admission, 69.2% presented with Rutherford Stage 5 ischemic ulcers. More than 70% were not receiving any prerevascularization antiplatelet therapy. According to the physical status classification, 95.6% were classified as ASA‐2 or ASA‐3.

**Table 1:** Baseline characteristics and clinical characteristics of the patients (*n* = 91).

Characteristic	Frequency (*n*)	Percentage (%)
Age (mean ± standard deviation)	74.01 ± 11.6	
Gender		
Male	75	82.4
Age group (years)		
40–59	10	11.0
60–79	47	51.6
≥ 80	34	37.4
Body mass index		
Underweight	20	22.0
Normal	63	69.2
Overweight	6	6.6
Obesity	2	2.2
Time of disease onset (month)		
< 1	22	24.2
1–6	58	63.7
6–12	10	11.0
> 12	1	1.1
Risk factors		
Hypertension	48	52.7
Diabetes	30	33.0
Cerebrovascular accident	10	11.0
Smoking	3	3.3
Previous vascular intervention	5	5.5
Heart failure	3	3.3
Smoking	2	2.2
Chronic kidney disease	1	1.1
Rutherford stage		
Stage 4	17	18.7
Stage 5	63	69.2
Stage 6	11	12.1
Prerevascularization antiplatelet therapy		
None	67	73.6
Aspirin	16	17.6
Clopidogrel	8	8.8
Physical status classification		
ASA‐1	0	0.0
ASA‐2	39	42.9
ASA‐3	48	52.7
ASA‐4	4	4.4

Abbreviation: ASA, American Society of Anesthesiologists.

**Figure 1:**
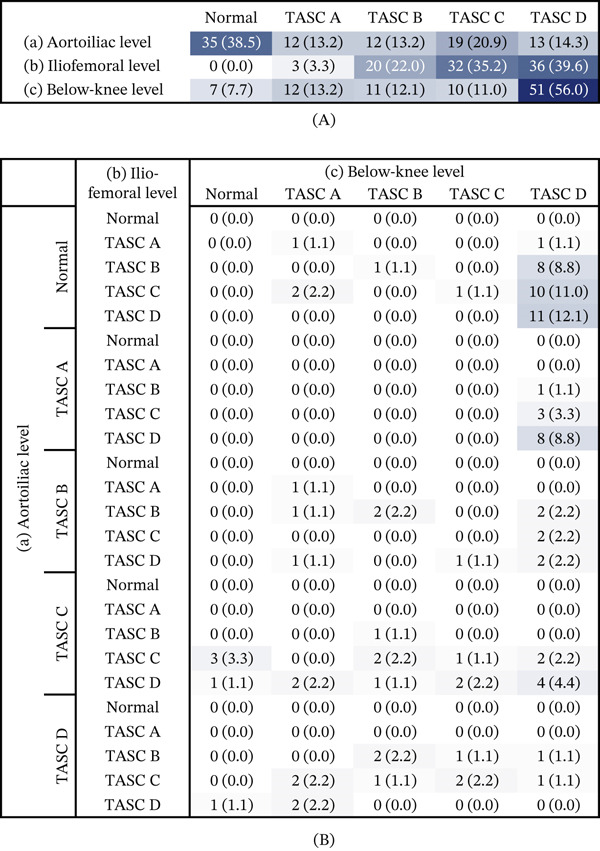
TASC II classification of peripheral arterial disease at different anatomical levels (*n* = 91). (A) Distribution of TASC II categories at separate aortoiliac, iliofemoral, and below‐knee levels. (B) Combined cross‐tabulation of TASC II categories across aortoiliac, iliofemoral, and below‐knee levels. Abbreviation: TASC, Trans‐Atlantic Inter‐Society Consensus.

Figure 1 illustrates the severity of arterial lesions according to the TASC II classification. Figure 1A describes the distribution of lesion severity across anatomical levels. At the aortoiliac level, 38.5% of patients had normal inflow or mild disease (TASC A), whereas 35.2% had complex lesions (TASC C/D). In contrast, lesions at the iliofemoral level were observed in all cases (100%), of which 35.2% were classified as TASC C and 39.6% as TASC D. The most severe involvement was noted at the below‐knee level, where 56.0% of patients were classified as TASC D.

The multilevel nature of arterial disease is detailed in Figure 1B. Among patients with a normal aortoiliac level, multilevel lesions were predominantly characterized by TASC D at the below‐knee level combined with TASC B (8.8%), TASC C (11.0%), or TASC D (12.1%) at the femoropopliteal level. In addition, 8.8% of cases showed concomitant TASC B at the aortoiliac level and TASC D at the below‐knee level. Meanwhile, 4.4% demonstrated TASC D lesions simultaneously at both the aortoiliac and below‐knee levels, with the iliofemoral level remaining normal.

### 3.2. Characteristics of Revascularization Treatments

Table 2 presents the characteristics of revascularization treatments. The majority of patients underwent the procedure under local anesthesia (87.9%, *n* = 80). More than half of the patients received multilevel revascularization (69.2%, *n* = 63), and the rate of complete revascularization was 27.5%. Initial arterial access via the contralateral femoral artery was most frequently used (45.1%, *n* = 41), compared with ipsilateral femoral artery access (35.2%, *n* = 32), whereas 19.8% (*n* = 18) required surgical exposure of the ipsilateral femoral artery for access. Iliofemoral balloon angioplasty was the most commonly performed procedure (91.2%, *n* = 83). Additional open revascularization procedures included femoral endarterectomy in 13 patients (14.3%) and Fogarty thrombectomy in 6 patients (6.6%). Postrevascularization antiplatelet therapy consisted of aspirin monotherapy in 47.2%, clopidogrel monotherapy in 19.8%, and dual antiplatelet therapy in 33.0%.

**Table 2:** Characteristics of revascularization treatments (*n* = 91).

Characteristic	Frequency (*n*)	Percentage (%)
Anesthesia method		
Volatile anesthesia	9	9.9
Total intravenous anesthesia	2	2.2
Local anesthesia	80	87.9
Revascularization method		
Complete revascularization	25	27.5
Partial revascularization	66	72.5
Single‐level revascularization	28	30.8
Multilevel revascularization	63	69.2
Initial arterial access		
Ipsilateral femoral artery	32	35.2
Contralateral femoral artery	41	45.1
Ipsilateral femoral artery surgery	18	19.8
Procedures		
Iliac artery balloon angioplasty	47	51.6
Iliofemoral balloon angioplasty	83	91.2
Anterior tibial artery angioplasty	9	9.9
Posterior tibial artery angioplasty	7	7.7
Peroneal artery angioplasty	11	12.1
Drug‐coated balloon angioplasty	2	2.2
Iliac artery stenting	33	36.3
Iliofemoral stenting	39	42.9
Additional open revascularization procedures		
Femoral endarterectomy	13	14.3
Fogarty thrombectomy	6	6.6
Wound care/minor amputation procedures		
Wound debridement, digit amputation	14	15.4
Chopart amputation	2	2.2
Postrevascularization antiplatelet therapy		
Aspirin	43	47.2
Clopidogrel	18	19.8
Dual antiplatelet therapy	30	33.0

**Figure 2:**
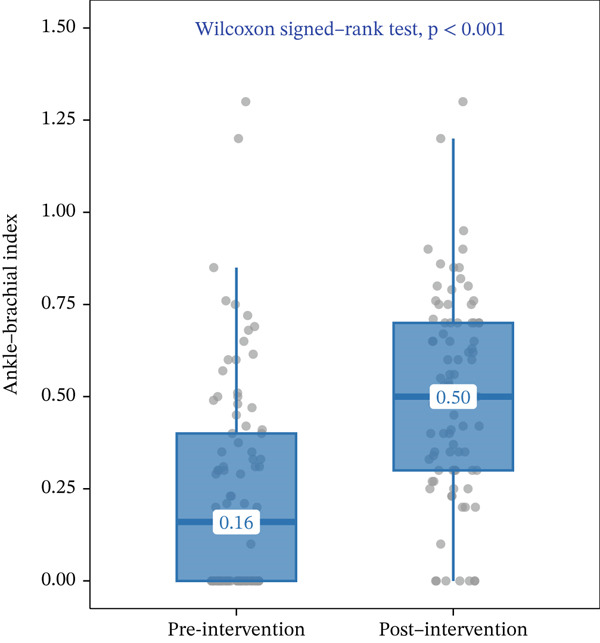
Improvement in the Ankle–Brachial Index before and after endovascular intervention (*n* = 91).

The ABI showed significant improvement after the intervention (*p* < 0.001). Before the intervention, the ABI values demonstrated a clearly left‐skewed distribution, with a median of 0.2 (0.0–0.4). However, after the intervention, the ABI increased and demonstrated a broader distribution, with a mean value of 0.5 ± 0.3 (Figure 2).

### 3.3. Midterm Clinical Outcomes

**Figure 3:**
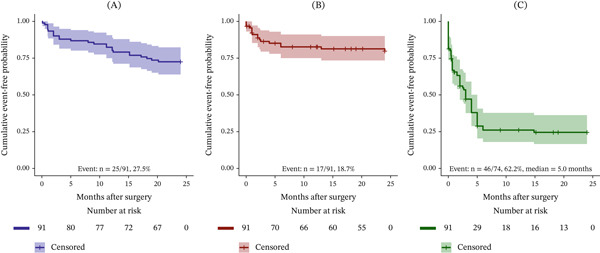
Kaplan–Meier estimates of clinical outcomes over 24 months post‐revascularization (*n* = 91). (A) Cumulative survival probability (all‐cause mortality). (B) Freedom from major limb amputation. (C) Cumulative wound healing rate in patients with tissue loss (*n* = 74).

The mean duration of follow‐up was 18.9 ± 8.3 months. Two patients were lost to follow‐up during the first 12 months (2.2%). During the follow‐up period from the date of intervention, 25 deaths were recorded (27.5%). A total of 17 patients (18.7%) underwent major amputation. The median time to wound healing was 5 months, and 46 patients (62.2%) achieved wound healing. Detailed outcomes are shown in Figure 3.

**Figure 4:**
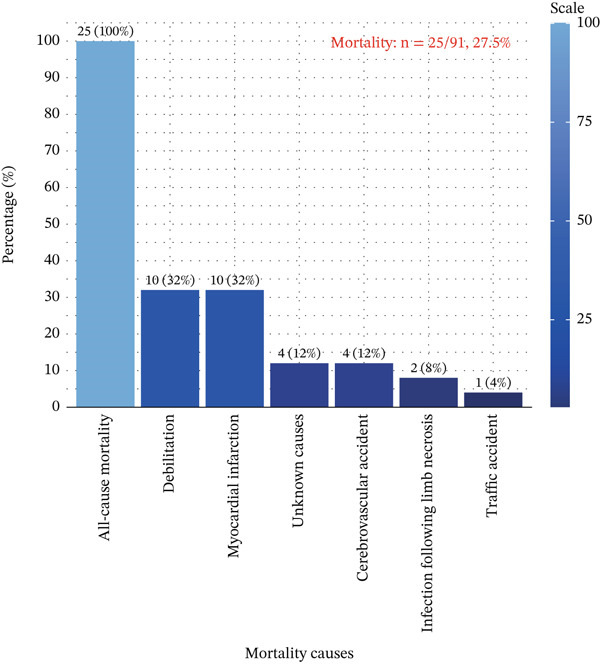
Midterm causes of post‐operative mortality (*n* = 91).

The all‐cause mortality rate within 24 months of follow‐up was 27.5% (25 patients), and the specific causes are presented in Figure 4. Debilitation and myocardial infarction were the two most common causes, each accounting for 32% (10 cases). Deaths due to unknown causes and cerebrovascular accident were each recorded in four patients (12%). Infection following limb necrosis was recorded in two cases (8%), and one death was due to a traffic accident (4%).

Factors associated with wound healing, all‐cause mortality, and major limb amputation within 24 months after revascularization are presented in Table 3. In multivariable logistic regression, age ≥ 75 years (aOR 0.32; 95% CI 0.12–0.86; *p* = 0.024) and NLR ≥ 3.5 (aOR 0.33; 95% CI 0.12–0.88; *p* = 0.027) were independently associated with lower odds of wound healing. In an exploratory posttreatment analysis, achievement of wound healing within 24 months was associated with lower odds of all‐cause mortality (aOR 0.16; 95% CI 0.05–0.49; *p* = 0.001) and major limb amputation (aOR 0.09; 95% CI 0.02–0.45; *p* = 0.003). Additional open revascularization procedures were also associated with lower odds of major limb amputation (aOR 0.10; 95% CI 0.01–0.98; *p* = 0.048).

**Table 3:** Factors associated with wound healing, all‐cause mortality, and major limb amputation within 24 months after revascularization (*n* = 91).

Covariates	Wound healing				Mortality				Limb amputation			
	cOR (95% CI)	*p* value	aOR (95% CI)	*p* value	cOR (95% CI)	*p* value	aOR (95% CI)	*p* value	cOR (95% CI)	*p* value	aOR (95% CI)	*p* value
Age ≥ 75 years	0.58 (0.35–0.96)	0.033	0.32 (0.12–0.86)	0.024	1.89 (0.73–4.88)	0.189	1.39 (0.48–4.02)	0.593	1.83 (0.61–5.48)	0.278	—	
Male	1.58 (0.50–4.98)	0.432	—		0.41 (0.13–1.25)	0.115	0.27 (0.07–1.05)	0.059	4.07 (0.50–33.16)	0.190	3.84 (0.38–38.68)	0.252
Hypertension	0.79 (0.30–2.08)	0.633	—		1.50 (0.59–3.82)	0.395	—		0.56 (0.19–1.64)	0.293	—	
Diabetes	0.47 (0.17–1.28)	0.138	0.44 (0.16–1.22)	0.115	1.53 (0.59–3.99)	0.381	—		0.82 (0.26–2.58)	0.730	—	
Rutherford 6	0.69 (0.19–2.51)	0.574	—		0.55 (0.11–2.75)	0.467	—		2.95 (0.75–11.53)	0.121	—	
Healed wound^a^	—		—		0.21 (0.08–0.57)	0.002	0.16 (0.05–0.491)	0.001	0.07 (0.02–0.34)	< 0.001	0.09 (0.02–0.45)	0.003
Adjunctive open surgery	2.32 (0.58–9.27)	0.236	—		—		—		0.21 (0.03–1.70)	0.143	0.10 (0.01–0.98)	0.048
Ratio N/L of 3.5 or greater	0.30 (0.11–0.82)	0.019	0.33 (0.12–0.88)	0.027	—		—		2.12 (0.72–6.45)	0.169	2.12 (0.56–8.04)	0.271
Complete revascularization	0.64 (0.23–1.75)	0.381	—		1.13 (0.35–3.60)	0.843	—		1.12 (0.35–3.60)	0.843	—	
TASC C/D at aortoiliac level	1.60 (0.56–4.57)	0.380	—		—		—		0.50 (0.15–1.70)	0.271	—	
TASC C/D at iliofemoral level	—		—		—		—		2.97 (0.63–14.14)	0.171	4.43 (0.77–25.42)	0.095
TASC D at below‐knee level	—		—		—		—		1.76 (0.52–5.95)	0.363	—	

Abbreviations: aOR, adjusted odds ratio; CI, confidence interval; cOR, crude odds ratio; N/L, neutrophil‐to‐lymphocyte; TASC, Trans‐Atlantic Inter‐Society Consensus.

^a^Wound healing was considered a posttreatment clinical outcome; its associations with all‐cause mortality and major limb amputation were exploratory and should not be interpreted as baseline prognostic effects.

## 4. Discussion

Endovascular revascularization is considered clinically successful in improving symptoms, with limb salvage rates of approximately 80% at 1 year and more than 60% at 5 years [12, 20]. The success of revascularization is commonly evaluated using three key outcomes: limb salvage, wound healing, and mortality. This single‐center retrospective case series is aimed at providing midterm outcomes of 91 patients who underwent endovascular revascularization for multilevel PAD (≥ 2 levels according to TASC II) accompanied by CLTI (Rutherford 4–6), with a follow‐up duration of 2 years. The analysis also evaluated factors associated with wound healing, limb amputation, and mortality to support risk stratification and optimize revascularization strategies in clinical practice.

The baseline characteristics of our cohort reflect an elderly population with multiple cardiovascular risk factors. Hypertension and diabetes were common comorbidities, consistent with previous studies of patients with CLTI [21–23]. Although smoking is one of the most important risk factors for patients with PAD, only 2% of patients in this study were current smokers. This proportion is substantially lower than the approximately 43% smoking prevalence reported in similar PAD populations in Asia. The relatively high mean age of patients in our study may explain this finding, as many patients might have stopped smoking after developing cardiovascular‐related conditions [24].

The primary goal of revascularization is to enhance blood flow to the limb in order to promote wound healing and optimize limb salvage. In our study, the midterm limb salvage rate was 81.3%. This rate varies when compared with previous studies, depending on the complexity of anatomical characteristics and the degree of systemic impairment. According to Casella et al. [25], the 2‐year limb salvage rate in patients with multilevel chronic lower limb arterial occlusive disease was 53.3% for those undergoing open surgical revascularization and 66.5% for those treated with endovascular revascularization. The authors attributed the relatively low limb salvage rate to the presence of below‐knee arterial lesions in all patients. Similarly, a study conducted across six cardiovascular centers in Japan [21], involving 406 patients with critical limb ischemia who underwent endovascular intervention, reported limb salvage rates ranging from 80% to 84% after 1–3 years of follow‐up. Although these findings are comparable to our results, an important difference in clinical context is that the study by Iida et al. focused exclusively on isolated below‐knee lesions, whereas our cohort represents a more challenging population, with 69.2% of patients having multilevel disease. However, in clinical practice, limb salvage alone does not always indicate successful management of CLTI. Persistent ischemic ulcers associated with chronic pain may continue to severely impair patients′ quality of life despite limb preservation [26].

Wound healing is also considered a patient‐centered outcome, alongside limb salvage and functional measures [27, 28]. In this study, the wound healing rate was 62.2% among 74 patients with tissue loss, with a median healing time of 5 months. This finding is consistent with a multicenter analysis by Okazaki et al. [26], which evaluated healing time and wound‐free survival after revascularization in patients with critical limb ischemia and tissue loss. They reported a 1‐year healing rate of 64.0%, which increased slightly to 69.7% at 2 years, with a mean healing time of 143.7 days (approximately 4.7 months). For patients with CLTI, revascularization represents only one component of the overall management strategy. Wound care is a critical element that requires a structured approach, multidisciplinary coordination, risk assessment, and efforts to prolong survival [29, 30].

After 2 years of follow‐up, the all‐cause mortality rate was 27.5%, and we observed that the midterm mortality rate varied considerably compared with both domestic and international studies. The Global Vascular Guidelines on the management of CLTI recommend that the estimated 2‐year mortality risk should be incorporated into treatment decision‐making for patients at moderate to high risk [22]. More recently, Zenunaj et al. proposed the GZ‐PAD score as a simple clinical risk model for predicting mortality after endovascular lower limb revascularization [31]. Although the present study was not designed to validate this score, our findings are consistent with the need for structured risk stratification in patients with CLTI, particularly in older patients and those with advanced tissue loss or systemic comorbidity [32]. In a retrospective study of 849 patients with CLTI treated predominantly with endovascular therapy [23], conducted to develop a risk stratification model for 2‐year mortality, the estimated mortality rate was 32.3%. Secemsky et al. [33] reported a survival rate of only 44.9% after a mean follow‐up of 2.72 years among patients who underwent femoral artery endovascular intervention. In addition, Lee et al. [34] evaluated revascularization outcomes in patients with chronic lower limb arterial occlusive disease in a multicenter study and reported a 1‐year mortality rate of 6.7%. The variation in mortality rates may be attributed to differences in the mean age of patients across study populations, as older age is associated with a higher risk of midterm mortality. Advanced age is often accompanied by a greater burden of comorbidities and physical frailty, which may impair neovascularization and delay wound healing [8]. The results of this study also showed that patients aged ≥ 60 years accounted for more than 90% of the cohort and those aged ≥ 75 years had a significantly lower likelihood of wound healing (aOR = 0.32; *p* = 0.024).

Although revascularization at our center improved limb perfusion, as reflected by a significant increase in ABI after intervention (*p* < 0.001), ABI has long been considered a useful clinical tool for assessing limb hemodynamics, with value in the diagnosis and monitoring of PAD, as well as in predicting cardiovascular risk and all‐cause mortality [2, 22, 35–37]. The independent association between a low ABI (≤ 0.90) and 10‐year cardiovascular mortality has been confirmed in a meta‐analysis of 16 community‐based studies [37], reflecting the burden of diffuse atherosclerosis throughout the vascular system. This may explain why the majority of deaths in the present study were due to cardiovascular causes, such as myocardial infarction and stroke. This finding reflects the burden of systemic atherosclerosis and the risk of systemic events. A similar pattern was reported by Hata et al. [23], in which infection and cardiovascular events accounted for the highest proportions, at 33.3% and 30.9%, respectively. Consistent with the advanced nature of PAD, CLTI is not confined to the limb but represents a progressive stage of diffuse atherosclerosis, with a higher risk of cardiovascular mortality compared with individuals without the disease [2].

In the management strategy for multilevel PAD accompanied by CLTI, risk stratification based on patient characteristics is as important as the assessment of lesion severity. After multivariable adjustment, age ≥ 75 years and an NLR of 3.5 or greater were independently associated with lower odds of wound healing. From a clinical perspective, wound healing is an important treatment milestone in patients with CLTI, reflecting restoration of perfusion after revascularization and effective infection control [38]. In the present study, wound healing within 24 months was associated with lower odds of all‐cause mortality and major limb amputation in an exploratory posttreatment analysis (aOR 0.16; *p* = 0.001 and aOR 0.09; *p* = 0.003, respectively). However, because wound healing is a postbaseline clinical outcome, this association should be interpreted cautiously and should not be considered a baseline prognostic factor. The possibility of reverse causation and immortal time bias should also be acknowledged. From a biological perspective, an NLR ≥ 3.5 may reflect the role of systemic inflammation in atherosclerosis and impaired tissue recovery. Several studies have also suggested that elevated systemic inflammatory markers are associated with an increased risk of cardiovascular disease [39–41]. Beyond biological barriers such as advanced age and systemic inflammation, patients who underwent additional open revascularization procedures had lower odds of major limb amputation compared with those treated without such procedures. Thus, in selected patients with complex multilevel PAD accompanied by CLTI, an endovascular‐first strategy combined with additional open revascularization may help optimize limb outcomes [42].

To our knowledge, these data represent one of the few studies providing midterm outcomes and evaluating associated factors in patients with multilevel PAD accompanied by CLTI, with a 2‐year follow‐up in the context of Vietnam, a developing country with limited healthcare workforce resources. The study focused on patient‐centered outcomes, reporting limb salvage, wound healing, and mortality simultaneously. Predictive factors were also identified, which may assist clinicians in recognizing high‐risk patients who require closer monitoring or more intensive supportive treatment. However, because selected patients underwent adjunctive open revascularization procedures, the findings should be interpreted as outcomes of an endovascular‐first revascularization strategy rather than purely endovascular treatment alone. In addition, treatment selection, including complete versus partial revascularization, was based on lesion anatomy, technical feasibility, and the treating surgeon′s clinical judgment; therefore, confounding by indication cannot be excluded. Although daily wound care is a crucial component in promoting wound healing, many patients are admitted with severe ischemic wounds accompanied by progressive infection, which may lead to cellulitis, abscess formation, osteomyelitis, gas gangrene, or necrotizing fasciitis, all of which make wound healing particularly difficult [43]. However, during the course of this study, we recognized a major limitation not only in this study but also in previous studies on PAD in Vietnam, namely, the inability to standardize postdischarge wound care, infection control, and follow‐up adherence, as most patients reside in distant provinces. The healthcare system in Vietnam is commonly organized in a hierarchical referral structure and is largely concentrated in major cities [44]. Even vascular surgery specialists are mainly available in large hospitals, such as the center where our study was conducted. Consequently, wound care for patients with PAD and CLTI in rural areas remains largely insufficient because of the shortage of healthcare professionals and limited medical facilities. Therefore, many patients living far from the hospital often choose to undergo amputation locally when wounds fail to heal for a prolonged period or when limb‐related events such as reocclusion occur, as they do not have the conditions for long‐term wound care and may be reluctant to return to urban hospitals because of distance or delayed detection. This situation may also influence the outcomes of the present study. In addition, the relatively small sample size and the single‐center retrospective design may introduce selection bias and limit the generalizability of the findings. Wound healing was assessed based solely on clinical evaluation; although this is common practice, we did not use advanced perfusion assessment methods such as skin perfusion pressure (SPP) measurement, which may better predict the likelihood of wound healing [31]. Antithrombotic therapy was another potential source of confounding. During the study period, postrevascularization antiplatelet therapy was not fully standardized, and the use of SAPT or DAPT reflected real‐world physician decision‐making and referral‐based follow‐up care. Therefore, residual confounding related to antithrombotic regimen, treatment duration, and adherence cannot be excluded.

## 5. Conclusion

An endovascular‐first revascularization strategy for multilevel PAD accompanied by CLTI is feasible, with a limb salvage rate exceeding 80% and more than half of patients with tissue loss achieving complete wound healing after 2 years. Midterm mortality remained high during follow‐up, although ABI improved significantly after revascularization. Patient characteristics such as advanced age, inflammatory status reflected by NLR ≥ 3.5, and the use of adjunctive open surgery were independent factors influencing treatment outcomes. Multimodal care, integrating revascularization, wound management, and optimization of medical therapy, is necessary to improve prognosis in patients with CLTI. Future studies should focus on individualized treatment strategies, and the incorporation of patient‐centered outcomes such as ambulatory function and quality of life may help guide optimization of care for patients with advanced multilevel PAD on a global scale.

## Funding

No funding was received for this manuscript.

## Conflicts of Interest

The authors declare no conflicts of interest.

## Data Availability

The data that support the findings of this study are available on request from the corresponding author. The data are not publicly available due to privacy or ethical restrictions.
